# Interpretable Short-Term Electrical Load Forecasting Scheme Using Cubist

**DOI:** 10.1155/2022/6892995

**Published:** 2022-02-08

**Authors:** Jihoon Moon, Sungwoo Park, Seungmin Rho, Eenjun Hwang

**Affiliations:** ^1^Department of Industrial Security, Chung-Ang University, Seoul, Republic of Korea; ^2^School of Electrical Engineering, Korea University, Seoul, Republic of Korea

## Abstract

Daily peak load forecasting (DPLF) and total daily load forecasting (TDLF) are essential for optimal power system operation from one day to one week later. This study develops a Cubist-based incremental learning model to perform accurate and interpretable DPLF and TDLF. To this end, we employ time-series cross-validation to effectively reflect recent electrical load trends and patterns when constructing the model. We also analyze variable importance to identify the most crucial factors in the Cubist model. In the experiments, we used two publicly available building datasets and three educational building cluster datasets. The results showed that the proposed model yielded averages of 7.77 and 10.06 in mean absolute percentage error and coefficient of variation of the root mean square error, respectively. We also confirmed that temperature and holiday information are significant external factors, and electrical loads one day and one week ago are significant internal factors.

## 1. Introduction

The increase in urban population has caused various problems, such as resource depletion, traffic congestion, and environmental pollution [1]. For the effective operation of complex urban systems, many municipalities and governments have been trying to transform existing cities into smart cities [2]. The smart city concept aims to improve the efficiency and security of urban infrastructure as well as the quality of life for its citizens [3]. For instance, smart cities can reduce GHG emissions by reducing traffic congestion and energy consumption and introducing technologies such as electric vehicles, energy storage systems (ESSs), and renewable energy (RE) [4]. In particular, improving the energy efficiency of buildings with ESSs and RE is an important issue for cities because building energy consumption is one of the main sources of GHG emissions [5]. Most smart city systems use recent technologies such as the Internet of Things and big data to implement various city services [6]. For instance, the energy efficiency of existing buildings can be improved using building energy management systems (BEMSs) [7].

A BEMS is a computer-aided tool that improves energy efficiency between the grid operator and consumers through bidirectional interaction [7, 8]. It collects and analyzes data related to electrical energy consumption to establish operational plans for building energy use [8]. On the demand side, a BEMS provides ways for customers to reduce or shift peak energy consumption and trade the remaining energy [9]. On the supply side, it serves as a tool for optimal allocation of RE, ESS, and demand response in electric utility grids [10]. Here, short-term load forecasting (STLF) has been widely used to determine the amount of power necessary for the reliable operation of electric utility grids from the next hour to the next week [9, 11]. It includes daily peak load forecasting (DPLF), total daily load forecasting (TDLF), hourly electrical load forecasting, and very short-term load forecasting (VSTLF) [12]. DPLF and TDLF are used to predict from one day to one week later as an essential procedure for unit commitment, energy trading, security analysis, and tight scheduling of outages and fuel supplies in power systems [13, 14].

Accurate STLF is challenging because typical electrical energy consumption has various patterns accompanied by uncertainties due to unforeseeable external factors [15]. Furthermore, when predicting electrical loads, it is necessary to adequately consider complex correlations between historical and current data [9]. Many studies have been conducted to achieve accurate STLF based on machine learning (ML) methods because they can properly extract implicit nonlinear relationships between input and output variables [16–19].  Table 1 briefly summarizes recent STLF models based on ML methods. For instance, Lee and Han [20] developed a day-ahead (DA) DPLF model using multiple linear regression (MLR). They collected daily peak electrical load data in South Korea from 2012 to 2016 through the Korea Power Exchange (KPX) and constructed an MLR model using the days of the week, seasons, average temperature, and historical loads from one day to one year before the prediction time point. The model achieved better predictive performance than the forecasting model of KPX, extreme learning machine, and autoregressive moving average (ARMA). Fan et al. [21] proposed a STLF model based on weighted K-nearest neighbor (KNN), called W-KNN. When constructing the KNN model, they considered the inverse of the Euclidean distance to give appropriate weights to each data point after selecting a value for K. In experiments using electrical load data from the National Electricity Market (Australia), their model outperformed KNN, ARMA, and artificial neural network (ANN) in predicting performance.

On the other hand, Dong et al. [22] developed an hourly electrical load forecasting model based on bootstrap aggregating (Bagging) for Chinese special days. They collected three years of hourly electrical load in Qingdao, China. To construct their STLF model, they defined a holiday variable, with 2 representing statutory days, 1 representing working days, and 0 representing bridging days, proximity days, and weekends. Their Bagging model showed better prediction performance than ANN and Bagging, which trained a holiday variable that included 1 for working days and 0 for holidays. Sun et al. [23] proposed an hourly electrical load forecasting model based on ANNs. They first collected hourly electrical load data of Tai'an City, Shandong Province, China, from 2016 to 2018. They then generated various input variables, considering timestamp, temperature, and historical electricity consumption to construct their STLF model. Their model showed a mean absolute percentage error (MAPE) of 4.11 and a mean absolute error of 33.88. Truong et al. [24] developed an additive ANN (AANN) model to predict building electrical energy consumption. They collected hourly electrical load data for one year from a residential building with an RE system and configured five input variables, such as days of the week, hours of the day, isolation, temperature, and historical electricity consumption, to train the AANN model. Their concept was based on a gradient boosting machine (GBM). Unlike GBM, which generally uses decision trees (DTs) as weak learners, the AANN trains iteratively by estimating an ANN as one weak learner and passing the remaining residuals back to other ANNs. The AANN model outperformed MLR, DT, ANN, and support vector regression (SVR) in predictive performance.

Recently, various hybrid STLF models based on two or more ML techniques have been proposed to derive better prediction performance than single ML-based STLF models. For instance, Fan et al. [25] proposed an SVR-based STLF model, namely, EMD-SVR-PSO-AR-GARCH, by hybridizing with empirical mode decomposition (EMD), particle swarm optimization (PSO), and autoregressive-generalized autoregressive conditional heteroscedasticity (AR-GARCH). They firstly decomposed an original electrical load data sequence into conventional intrinsic mode functions (IMFs) and residuals using the EMD. Then, they used SVR-optimized PSO and AR-GARCH to fit IMF1 and other IMF components and residuals, respectively. Finally, they obtained the final prediction value by integrating and fitting the prediction values of the models. Their model outperformed ARMA, AR-GARCH, SVR, and others in predictive performance. Zhang et al. [26] developed a hybrid STLF model, called VMD-SR-SVRCBCS, using variational mode decomposition (VMD) and self-recurrent (SR)-SVR by optimizing the parameters through the cuckoo bird search process of the cuckoo search algorithm (CBCS). They performed data preprocessing using the VMD to obtain more accurate IMFs and applied SR-SVRCBCS to model each decomposed IMF for more accurate forecasting results. Here, the SR mechanism, inspired from the combination of Jordan's and Elman's recurrent neural network, was used to learn more recurrent information from the hidden layer values at the previous time point concerning the outcomes of the SVRCBCS models. Their model outperformed single ML models such as autoregressive integrated moving average (ARIMA), seasonal ARIMA, ANN, and SVR in predictive performance.

However, because the decision-making process inside most of these models mentioned above is opaque (i.e., a black box), forecasting results derived from these models cannot be entirely accepted and utilized. Therefore, their interpretation has been another challenging task [32]. Recently, interpretability methods in ML have attracted increasing attention for constructing accurate and interpretable forecasting models [33, 34]. Here, “interpretable” means that the user can understand how the model employs the input variables to make predictions [33]. The variable importance (also referred to as feature importance) measure is the basis for enhancing the interpretability of a model [34]. Several studies have used the variable importance measure to confirm the most significant factors in STLF. Bouktif et al. [27] proposed a long short-term memory (LSTM) network-based STLF model using Metropolitan France's electrical load data. They used a genetic algorithm to optimize the hyperparameter tuning of the LSTM model. They also confirmed that the historical load was the most significant input variable for model training using the variable importance of extra trees (ETs). Their LSTM model outperformed ridge regression, KNN, RF, GBM, ANN, and ET. Wang et al. [28] proposed an hourly electrical load forecasting model based on RF. They configured ten input variables representing weather, occupancy, and time-related data and constructed a forecasting model for each academic semester. They predicted the electrical load of two educational buildings and identified the most influential variables that differed by semester. The RF model performed better than the DT and SVR models.

Ruiz-Abellón et al. [29] developed four 48-hour-ahead electrical load forecasting models using hourly electrical load data collected from the Technical University of Cartagena in Spain. They used Bagging, RF, conditional RF (CRF), and XGB as tree-based ensemble methods to construct the forecasting models. They also described the essential input variables for training each forecasting model through variable importance. In the experiments, the XGB model achieved better prediction performance than Bagging, RF, and CRF. Abbasi et al. [30] proposed a 30-minute-interval electrical load forecasting model based on XGB. They used variable importance to extract input variables from the historical load during a week and confirmed that the historical loads close to the prediction time point and from a week before the prediction time point had high importance for the model construction. Subsequently, they constructed the XGB-based forecasting model using the extracted input variables. The XGB model exhibited a MAPE of 10% and an accuracy of 97%. Zhang et al. [31] proposed a TDLF model based on K-means clustering and categorical boosting (CatBoost). They collected total daily electrical load data from Yangzhong High-Tech Zone in China and executed K-means clustering to group industrial customers with similar load features into the same cluster. They then constructed a CatBoost-based TDLF model for each cluster and showed the variable importance of the CatBoost model. Their proposed model outperformed ARMA, LSTM, GBM, and CatBoost in predictive performance.

Despite these efforts, there are still limitations. For instance, when a forecasting model based on these methods is constructed using conventional time-series forecasting model evaluation [35], we can determine the essential input variables by analyzing the variable importance in the model constructed from the training dataset. However, conventional time-series forecasting model evaluation performs unsatisfactorily when there is a significant gap between the training set period and the test set period [14]. This makes it difficult to ensure confidence in the decision-making process of the model. In addition, most of the studies were conducted mainly on STLF with high time resolution, such as hourly or subhourly intervals, for DA energy planning. Therefore, further studies are needed on the quantitative DPLF and TDLF for DA and weak-ahead (WA) energy planning. Furthermore, although a Cubist regression model has shown excellent performance in time-series forecasting [36–38], its use for STLF has rarely been reported [38].

To address these issues, this study proposes a robust interpretable short-term electrical load forecasting model for accurate DPLF and TDLF. To this end, we collected five electrical load datasets from two commercial buildings and three educational building clusters. We configured various input variables that highly correlate with DPLF and TDLF. Then, we constructed STLF models using Cubist and time-series cross-validation (TSCV) to achieve high accuracy. The main contributions of this paper are as follows:

1. We use electrical load data from two public buildings and three building clusters with different purposes to predict building-level electrical energy consumption, which has more complex patterns.
2. We configure different input variables to predict both DPLF and TDLF by considering both DA and WA energy planning for effective BEMS operation.
3. When a forecasting model is constructed for WA forecasting, we obtain multistep-ahead forecasting of all prediction time points (from one day to seven days) to compensate for uncertainty.
4. To address the data shortage problem and reflect current electrical load trends and patterns, we use the cross-validation procedure based on a rolling forecasting origin.
5. We compare our proposed model with other popular statistical and machine learning methods in terms of four forecasting types: DA-DPLF, DA-TDLF, WA-DPLF, and WA-TDLF.
6. We perform an in-depth analysis of which input variables are the most important factors in electrical load forecasting for each dataset by using the variable importance of the proposed model.

The remainder of this paper is organized as follows.  Section 2 describes the data preprocessing used to configure different input variables for forecasting types and presents the process of constructing the proposed model. In Section 3, we analyze the experimental results to demonstrate the superiority of the proposed model. Finally, we conclude our study and present the directions for future research in Section 4.

## 2. Materials and Methods

**Figure 1:**
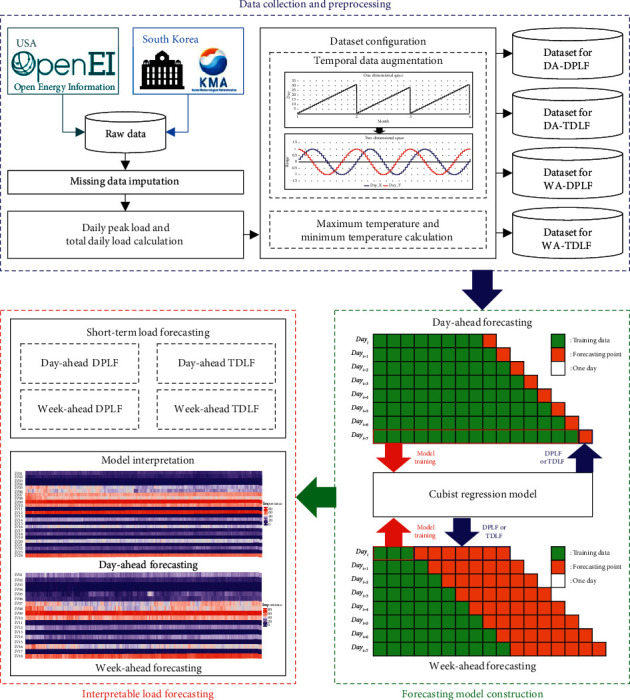
Architecture of interpretable short-term electrical load forecasting model (DA: day-ahead, WA: week-ahead, DPLF: daily peak load forecasting, and TDLF: total daily load forecasting).

In this section, we describe in detail the data preprocessing and forecasting model construction.  Figure 1 illustrates the overall flowchart of the proposed method.

### 2.1. Data Collection and Preprocessing

**Figure 2:**
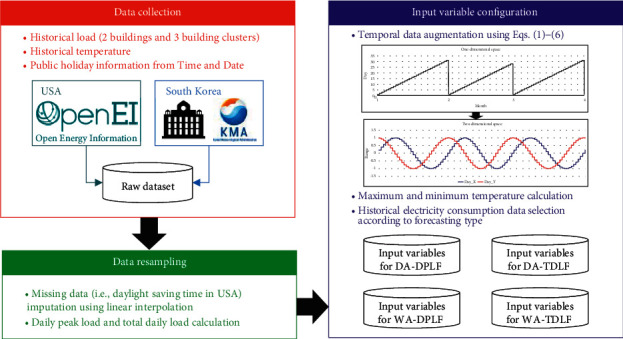
Data preprocessing for Cubist modeling (DA: day-ahead, WA: week-ahead, DPLF: daily peak load forecasting, and TDLF: total daily load forecasting).

In this section, we first present a data resampling process to construct data suitable for forecasting purposes. Then, we describe the process of configuring the input variables according to the purpose (i.e., DA and WA forecasting) using timestamps, weather, and historical load information.  Figure 2 illustrates the framework of the data preprocessing for constructing a Cubist model.

We collected electrical load datasets from five different types of buildings or building clusters, as summarized in Table 2. We first collected publicly available datasets from two buildings in Richland, WA, USA [39, 40]. The datasets consist of three years' worth of information, including the hourly electrical load, the hourly outdoor temperature, and the corresponding timestamps. We filled in the missing values (i.e., daylight saving time in North America) in both datasets using linear interpolation. Because the dataset contains the hourly electrical load data for a day (24 rows), we calculated the maximum load value and the sum of all load values for each day for DPLF and TDLF, respectively.

In addition, we collected typical 15-minute-interval electrical load datasets from three clusters of buildings at a private university in Seoul, South Korea [41]. The dataset collection period was three years. The first cluster comprised 16 residential buildings, and the electrical load had a residential pattern. The second cluster consisted of 32 academic buildings, including the main hall, library, classrooms, and offices. The third cluster contained five science and engineering buildings. This cluster exhibited much higher electrical loads per building than the other clusters, mainly due to the various experimental equipment and devices in the laboratories. In South Korea, the daily peak load of the building was calculated by multiplying the highest value among the 15-minute-interval electrical load used per day by 4. We took this into account when calculating the daily peak electrical load for DPLF. Likewise, we took the sum of 96 values per day to perform TDLF.

Tables 3 and 4 provide some statistics on daily peak loads and total daily loads for the five datasets, respectively. Weather conditions and holiday information are closely related to electrical load [42]. Therefore, we used these data to configure the input variables for Cubist modeling. Because we performed two types of forecasting, namely, DA and WA forecasting, we used different input variable configurations for each type of forecasting.

Time is another important factor for electrical loads. We considered various temporal variables, such as months, days, and days of the week. However, these variables cannot reflect periodic information when applied to forecasting models. For instance, 31 December and 1 January are temporally contiguous, yet, the range of both values in sequence form is 30 and 11 as day and month, respectively. Hence, we represented them as continuous data in the two-dimensional (2D) space to reflect their periodicity using equations (1)–(6) [41, 42]. Here, the variable for seven days of the week is defined as 1 to 7 from Monday to Sunday, according to international standard ISO 8601. LDM_month_ represents the last day of the month to which the day belongs (e.g., January: 31, February: 28 or 29, March: 31, and so on). Consequently, we used six input variables to describe the date and time of the prediction time points.

(1) $$\begin{array}{c} {\text{Month}}_{\text{x}}=\sin\left(\left(\frac{360}{12}\right)\times \text{Month}\right), \end{array}$$

(2) $$\begin{array}{c} {\text{Month}}_{\text{y}}=\cos\left(\left(\frac{360}{12}\right)\times \text{Month}\right), \end{array}$$

(3) $$\begin{array}{c} {\text{Day}}_{\text{x}}=\sin\left(\left(\frac{360}{{\text{LDM}}_{\text{month}}}\right)\times \text{Day}\right), \end{array}$$

(4) $$\begin{array}{c} {\text{Day}}_{\text{y}}=\cos\left(\left(\frac{360}{{\text{LDM}}_{\text{month}}}\right)\times \text{Day}\right), \end{array}$$

(5) $$\begin{array}{c} \text{Day}\_\text{of}\_\text{the}\_{\text{Week}}_{\text{x}}=\sin\left(\left(\frac{360}{7}\right)\times \text{Day}\_\text{of}\_\text{the}\_\text{week}\right), \end{array}$$

(6) $$\begin{array}{c} \text{Day}\_\text{of}\_\text{the}\_{\text{Week}}_{\text{y}}=\cos\left(\left(\frac{360}{7}\right)\times \text{Day}\_\text{of}\_\text{the}\_\text{week}\right). \end{array}$$

To verify the validity and applicability of the 2D representation, we computed several regression statistics on the electrical loads in one-dimensional (1D) space, consisting of three temporal variables (i.e., months, days, and days of the week), and 2D space, as shown in Tables 5 and 6. Here, the residual standard error (RSE), multiple R-squared, and adjusted R-squared were calculated using equations (7)–(9).

(7) $$\begin{array}{c} \text{RSE}=\sqrt{\frac{1}{\text{n}-2}\overset{\text{n}}{\underset{\text{t}=1}{\sum }}{\left({\text{y}}_{\text{t}}-\hat{{\text{y}}_{\text{t}}}\right)}^{2}}, \end{array}$$

(8) $$\begin{array}{c} {\text{R}}^{2}=1-\frac{{\sum \left({\text{y}}_{\text{t}}-\hat{{\text{y}}_{\text{t}}}\right)}^{2}}{{\sum \left({\text{y}}_{\text{t}}-\bar{\text{y}}\right)}^{2}}, \end{array}$$

(9) $$\begin{array}{c} \text{Adjusted}\_{\text{R}}^{2}=1-\frac{\left(1-{\text{R}}^{2}\right)\left(\text{n}-1\right)}{\text{n}-\text{p}-1}, \end{array}$$

where y_t_ and $\hat{{\text{y}}_{\text{t}}}$ represent the actual and estimated values, respectively, at the time t. $\bar{\text{y}}$ represents the mean of the actual values, and n and p indicate the number of observations and variables, respectively. From the tables, we can observe that the 2D representation exhibits correlations more effectively than the 1D representation.

Typical electrical load patterns are different on weekdays and holidays, depending on the type of building [16, 40]. To reflect this, holiday information was also used as an input variable of the forecasting model. We collected holiday information by country from https://www.timeanddate.com [43]. The holidays included Saturdays, Sundays, and public holidays. We used one-hot encoding (i.e., “1” for the relevant data and “0” otherwise) as a nominal scale. Hence, we used seven time factors as input variables at the prediction time point.

**Figure 3:**
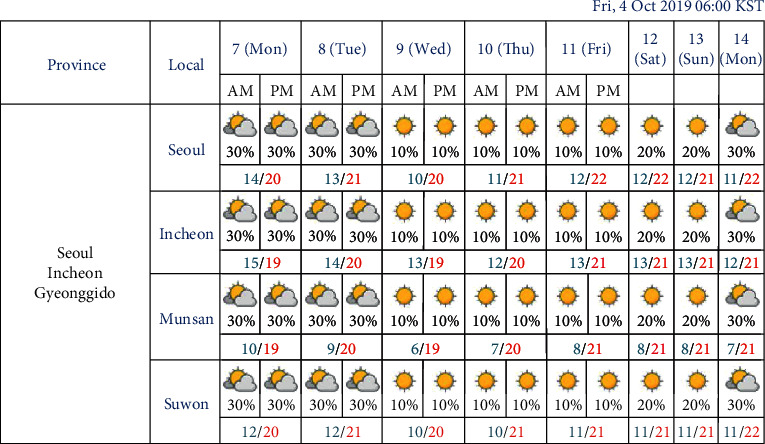
Example of mid-term forecast provided by the KMA.

In general, the electrical load increases in the summer and winter due to the heavy use of air-conditioning and electrical heating appliances, respectively [13, 44]. Here, the most influential factor in the electrical load is temperature [13]. In this study, we focused on temperature-related variables, such as the daily maximum, average, and minimum temperatures [44]. In the case of Buildings 1 and 2, because the datasets we collected also included the outdoor temperature along with the electrical load, we calculated the daily maximum, average, and minimum temperatures (in the Fahrenheit scale). For Clusters 1 to 3, we first collected hourly temperature data using regional synoptic meteorological data provided by the Korea Meteorological Administration (KMA) and collected by the Seoul Meteorological Observatory located about 6 km from the university campus. As in Buildings 1 and 2, we calculated the daily maximum, average, and minimum temperature in Celsius. In South Korea, the KMA provides weather forecasts, including short and mid-term forecasts, for the most significant regions. The KMA mid-term forecast service provides the maximum and minimum temperatures from day 3 to day 10, as shown in Figure 3.

Historical electrical load data are particularly important in electrical load forecasting because they exhibit the trend of recent electrical loads [17, 45]. To reflect the recent trend in the prediction, we used the daily peak and total daily loads of the previous seven days as input variables for the DPLF and TDLF models, respectively. Because the electrical load trends of weekdays and holidays can differ, we added a holiday indicator to indicate whether the day was a holiday [46]. We used a total of 24 input variables to build the DA-DPLF and TDLF models.

Now, we describe the configuration of the input variables for the WA-DPLF and TDLF models. First, we used the same time and temperature variables as in the DA forecasting models. However, the daily peak and total daily loads from the previous day to the sixth day before the forecast date are unknown when considering WA forecasting. To compensate for this, we configured the input variables using the daily peak and total daily loads and holiday indicators of the same day of the previous four weeks for the WA-DPLF and TDLF, respectively [46]. Therefore, we used a total of 18 input variables to construct our WA electrical load forecasting models.  Table 7 presents all the input variables that we considered for DA and WA forecasts.

### 2.2. Forecasting Model Construction

Variable importance is a technique that assigns scores to input variables based on the relative importance of each variable for accurate prediction [34]. Variable importance scores can be evaluated for both classification and regression problems. Variable importance scores play an essential role in constructing forecasting models because they can provide insight into the dataset or the model supported by the dataset [47]. For instance, relative scores can highlight which input variables have the greatest or least effect on the output variable. These scores can be interpreted by domain experts and used as a reference for collecting more or different data. Because most variable importance scores are calculated by forecasting models that fit the dataset, these scores can be calculated for the model interpretation. In addition, analyzing variable importance can offer suggestions to improve the efficiency and effectiveness of the forecasting model through dimensionality reduction and feature selection [48].

**Figure 4:**
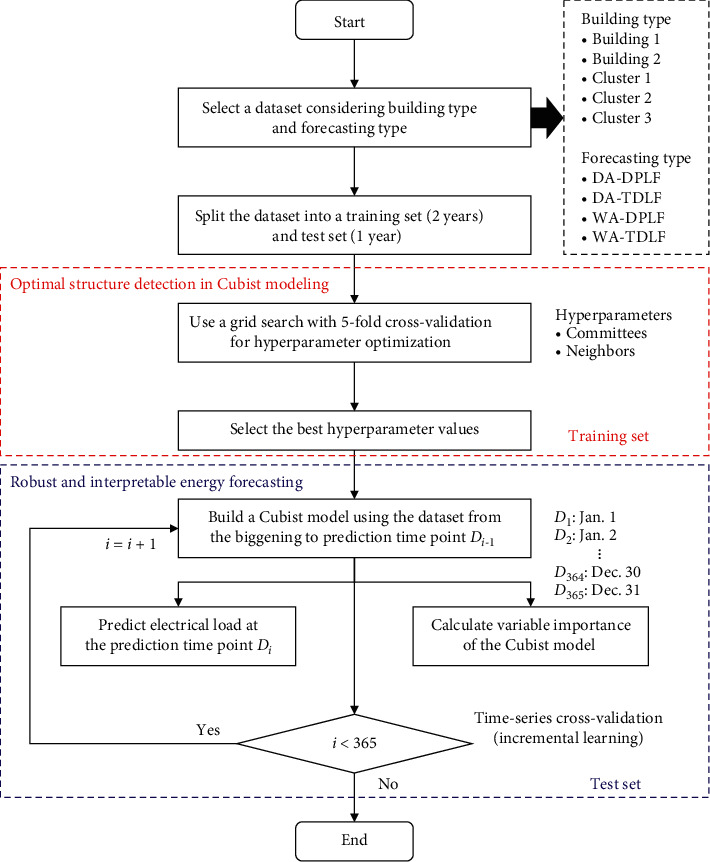
Flowchart of interpretable electrical load forecasting based on Cubist modeling (DA: day-ahead, WA: week-ahead, DPLF: daily peak load forecasting, and TDLF: total daily load forecasting).

To date, the R language has been widely used for data cleansing, preparation, and analysis [49]. To facilitate accessibility, we also adopted multiple R packages, including variable importance evaluation functions. To calculate variable importance measures in the R environment, we used Cubist, a regression tree-based model, because it provides a balance between interpretability and predictive power [36].  Figure 4 exhibits the flowchart of interpretable electrical load forecasting based on Cubist modeling. Cubist was developed based on Quinlan's M5 model tree [48, 50]. The Cubist method generates a series of “if-after-after” rules. Each rule holds a linked multivariate linear model. As long as the covariate set meets the rule conditions, the corresponding model is used to compute the predicted value. The Cubist output includes variable usage statistics and provides the percentage of times each variable was adopted in a condition and/or a linear model.

The general concept of a Cubist regression model can be explained as follows.

1. While the tree is growing, many leaves and branches grow.
2. Branches can be considered a series of “if-then” rules, whereas terminal leaves remain a connected multivariate linear model.
3. Assuming that the covariate set complies with the rule conditions, the relevant model is used to measure the predicted value.

The Cubist model sequentially develops a series of trees with adjusted weights and strengthens them with training committees (usually one or more), similar to the “boosting” method. The number of neighbors in the Cubist model is used to correct rule-based predicted values, and the final predicted value denotes a function of all the linear models from the initial node to the terminal node. The percentages displayed in the Cubist output reflect all the models related in the predicted value rather than the terminal models displayed in the output. The variable importance used here is a linear combination of the rule condition usage and model [50].

When constructing a forecasting model, datasets are usually divided into a training set and a test set, and then the forecasting model is built using the training set and verified using the test set [35]. However, conventional time-series forecasting model evaluation could exhibit unsatisfactory prediction performance when there is a significant gap between the training set period and the test set period. Additionally, when the dataset is insufficient, it is challenging to obtain satisfactory prediction performance using a small amount of training data [19].

**Figure 5:**
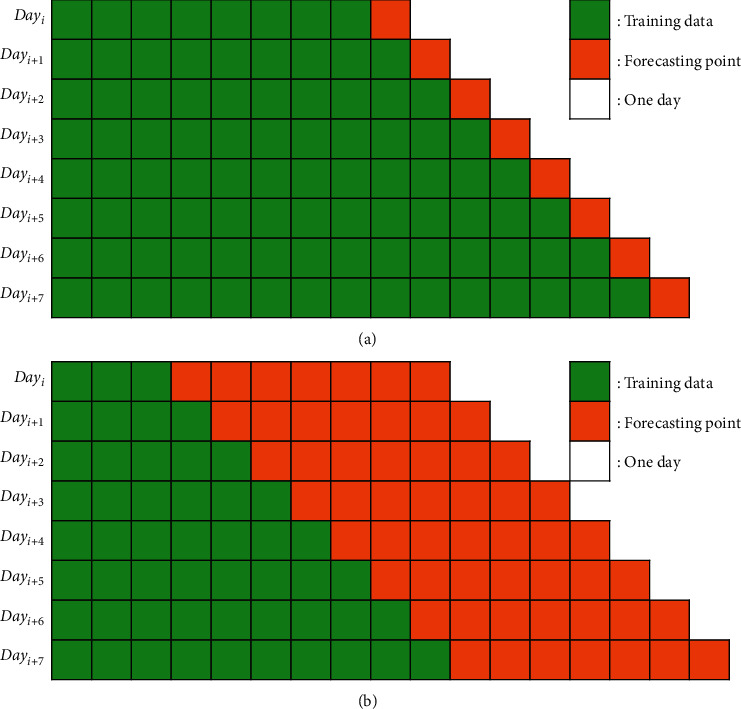
. Time-series cross-validation for day-ahead forecasting and week-ahead forecasting. (a) Day-ahead forecasting. (b) Week-ahead forecasting.

To solve these problems, we utilized TSCV based on a rolling forecasting origin [51]. TSCV focuses on a single or several forecast horizons for each test set. In this study, we used several different training sets, each containing one or more observations not used in the previous training set, depending on the scheduling period. To perform DPLF and TDLF, we took the test sets one day after the current time and a week after the current time, as shown in Figure 5. We evaluated the forecasting model performance by calculating the prediction accuracy at each time point and then calculated their average value.

Therefore, it is possible to solve the data shortage problem because more data can be used over time than in the conventional time-series forecasting model evaluation. We can also expect satisfactory prediction performance because it can adequately reflect recent electrical load patterns and adjust the weights of the input variables in the forecasting model. Here, we presented interpretable electrical load forecasting results by calculating the importance of the variables for each training set in the model.

## 3. Results and Discussion

In this section, we first introduce metrics to compare the prediction performance of the forecasting models and describe the experimental design and results in detail. We also present the results of several statistical tests to prove the validity of our experiment. We exhibit the interpretable short-term electrical load forecasting using the proposed model. Finally, we discuss experimental procedures.

### 3.1. Experimental Design

The quantitative experiments were conducted with an Intel® CoreTM i7-8700k CPU with 32 GB DDR4 RAM. We performed the input variable configuration and forecasting model construction in RStudio (v. 1.1.453) with R (v. 3.5.1). We used three years of electrical load data from 2009 to 2011 for Buildings 1 and 2 and from 2016 to 2018 for Clusters 1 to 3, respectively. We divided the dataset into training (in-sample) and test (out-of-sample) sets in an approximate proportion of 2 : 1. For Buildings 1 and 2, we confirmed that the electrical load from November to December 2011, the out-of-sample period, was higher than that for the remaining days. The R random number generator seed was set to 1234 for all methods.

To evaluate the predictive performance of forecasting models, we used the MAPE and coefficient of variation of the root mean square error (CVRMSE). MAPE and CVRMSE values show the accuracy as a relative percentage error. Hence, they are easier to understand than other well-known metrics, such as the mean absolute error, mean square error, and root mean square error [40, 52]. The MAPE measures the prediction accuracy for constructing fitted time-series values in statistics, specifically in trend estimation. The CVRMSE is used to aggregate the residuals into a single measure of predictive ability and is more useful when significant errors are particularly undesirable. The lower the MAPE and CVRMSE values, the better the forecasting model's predictive performance. However, it is known that the MAPE and CVRMSE increase significantly when the actual value tends to zero [35, 52]. The MAPE and CVRMSE are calculated using (10) and (11), respectively, where y_t_ and $\hat{{\text{y}}_{\text{t}}}$ are the actual and forecasted values at time t, respectively, $\bar{\text{y}}$ is an average of the actual values, and n is the number of observations.

(10) $$\begin{array}{c} \text{MAPE}=\frac{100}{\text{n}}\sum_{\text{t}=1}^{\text{n}}\left|\frac{{\text{y}}_{\text{t}}-\hat{{\text{y}}_{\text{t}}}}{{\text{y}}_{\text{t}}}\right|, \end{array}$$

(11) $$\begin{array}{c} \text{CVRMSE}=\frac{100}{\bar{\text{y}}}\sqrt{\frac{\sum_{\text{t}=1}^{\text{n}}{\left({\text{y}}_{\text{t}}-\hat{{\text{y}}_{\text{t}}}\right)}^{2}}{\text{n}}}. \end{array}$$

To demonstrate the validity of the proposed model, we considered a total of 12 machine learning methods including MLR, partial least squares (PLS), multivariate adaptive regression splines (MARS), KNN, SVR, DT, Bagging, RF, GBM, XGB, and CatBoost. Most machine learning methods have hyperparameters that can influence model performance. To further improve their performance, it is critical to tune the hyperparameters effectively. To find the optimal hyperparameters, we performed 10-fold cross-validation, all on the training set.  Table 8 describes the hyperparameters for each method, the R package used, the references where details about their optimal value can be found, and the hyperparameter ranges.

For DA forecasting, we also considered the MLR model in [20], which achieved better prediction performance than KPX's forecasting model, as a baseline model in the evaluation of the proposed model. The MLR model adapts a rolling procedure using a dataset from one day to one year before the prediction time point and is specified in the following equation:

(12) $$\begin{array}{c} {\text{Y}}_{\text{D}}=\beta_{0}+\beta_{1}{\text{Y}}_{\text{D}-1}+\beta_{2}{\text{Y}}_{\text{D}-2}+\beta_{3}{\text{Y}}_{\text{D}-7}+\beta_{4}{\text{W}}_{\text{D}}+\beta_{5}{\text{S}}_{\text{D}}+\beta_{6}{\text{T}}_{\text{D}}+\beta_{7}{\text{S}}_{\text{D}}{\text{T}}_{\text{D}}+\epsilon , \end{array}$$

where Y_D_ is the expected daily peak or total daily electrical load at day D; Y_D−1_,  Y_D−2_, and Y_D−7_ are historical loads before one day, two days, and one week, respectively; W_D_, which represents the day of the week at day *D*, is a categorical variable and consists of seven categories from 1 (Monday) to 7 (Sunday); S_D_ is a categorical variable for season and consists of six categories from 1 (Jan. and Feb.) to 6 (Nov. and Dec.); T_D_ is the average temperature at day D; S_D_T_D_ is a quadratic variable to reflect appropriate weather information according to the season; and *β*_0_, *β*_*i*_, and *ϵ* are the constant term, the slope coefficient of the ith independent variable, and the error term, respectively.

### 3.2. Experimental Results

Tables 9–12 present the MAPE and CVRMSE results of DA-DPLF and TDLF, respectively, through conventional time-series forecasting model evaluation, also known as holdout, and TSCV. Tables 13–16 present the MAPE and CVRMSE forecasting results for DPLF and TDLF, respectively. In Tables 9–16, we demonstrate that the Cubist method produced lower MAPE and CVRMSE values than any other forecasting method that we considered. We also exhibit the prediction performance for each time point through holdout and TSCV. In most cases, TSCV exhibited better prediction performance than holdout, except for PLS, where the input variable weight was adjusted in the forecasting model to reflect the recent electrical load pattern.

We made the following five observations from the experiments:

1. The day-ahead electrical load forecasting models exhibited better prediction performance than the week-ahead electrical load forecasting models.
2. The week-ahead electrical load forecasting models exhibited a lower prediction performance as the prediction time moved further from the current time.
3. Despite the same time point, the day-ahead electrical load forecasting models showed better prediction performance than the first prediction time point of the week-ahead electrical load forecasting models.
4. The total electrical load forecasting models displayed higher prediction performance than the peak electrical load forecasting models.
5. The prediction accuracy for Clusters 1–3 was higher than the prediction accuracy for Buildings 1 and 2.

In general, the electrical load pattern of buildings or building clusters could change from a variety of causes, and hence the further apart the current and forecast times, the higher the uncertainty [9]. Therefore, both the DA and the first prediction time point of the WA electrical load forecasting models yielded more accurate predictions because they modeled electrical load patterns of the day before using TSCV. In addition, because the DA electrical load forecasting models utilized the electrical load of the day before as an input variable, they exhibited more accurate prediction performance than the first prediction time point of the WA electrical load forecasting models. Hence, we confirmed that the electrical loads during the week were more significant input variables than the electrical loads of the same days of the week. Overall, we also confirmed that the forecasting models displayed low prediction accuracy when the electrical load was close to zero. From November to December 2011 (out-of-sample), Buildings 1 and 2 showed a sudden higher electrical load than that on other days. Thus, the electrical load forecasting models had a large prediction error because they were not trained on these electrical loads.

To find the best forecasting method, we ranked them by considering all the TSCV performance metrics for each building or cluster. We then calculated the average rank using the rank for all buildings and clusters for the DA-DPLF and TDLF and WA-DPLF and TDLF for each method.  Table 17 presents the ranks for each method for each performance metric and the average ranks. We confirmed that the Cubist method exhibited the best rank in the table. In addition, we demonstrated that the proposed Cubist model outperformed the MLR model in all aspects of DA forecasting, as shown in Table 18.

### 3.3. Statistical Verification

To demonstrate the validity of the proposed method, we conducted three statistical tests. The paired sample *t*-test was used to demonstrate the effectiveness of TSCV, and the Wilcoxon signed-rank and Friedman tests were used to confirm the difference in prediction performance between the proposed model and the other models. Here, the *p* value gives the probability of observing test results under the null hypothesis. The cutoff value for determining statistical significance is usually a value of less than 0.05, which corresponds to a 5% or lower chance of obtaining a result like the one observed if the null hypothesis is correct.

The paired sample *t*-test compares two means of the same individual, object, or related units. The test objective is to discover whether there is statistical evidence that the mean difference between a pair of observations for a critical result is significantly different from zero. Typical applications of the paired sample *t*-test include case-control studies or repeated-measurement designs. Because PLS performed poorly for all forecasting tasks in TSCV, we performed the paired sample *t*-test considering all the holdout and TSCV values for each method except PLS for MAPE and CVRMSE.  Table 19 shows that the MAPE and CVRMSE *p* values were both less than 0.05. Therefore, we can confirm the validity of the TSCV used in this study.

The Wilcoxon signed-rank test [58, 59] is used to confirm the null hypothesis to determine a significant difference between the two models. In contrast, the Friedman test [58] is a multiple-comparison test that aims to identify significant differences between three or more forecasting models. To verify these test results, we used all the MAPE and CVRMSE values (the DA-DPLF and TDLF and the WA-DPLF and TDLF) for each forecasting method. The Wilcoxon signed-rank test results with a significance level of 0.5 and the Friedman test results are shown in Table 20. Because the *p* value in all cases is below the significance level, the proposed model is superior to the other models.

### 3.4. Model Interpretation

**Figure 6:**
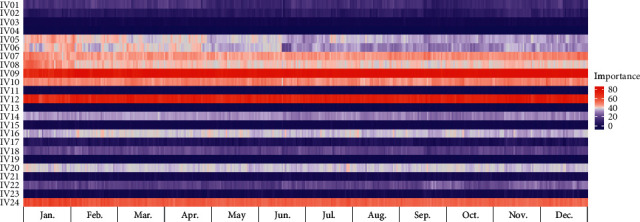
Example of variable importance for DA-DPLF (Building 1).

**Figure 7:**
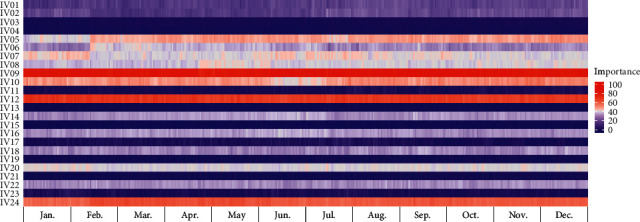
Example of variable importance for DA-TDLF (Building 1).

**Figure 8:**
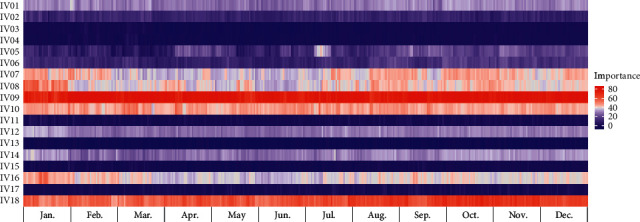
Example of variable importance for WA-DPLF (Building 1).

**Figure 9:**
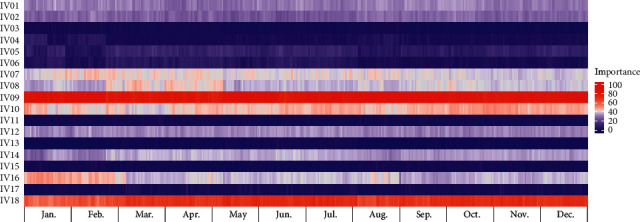
Example of variable importance for WA-TDLF (Building 1).

We determined the variable importance of the proposed model at each test point (one day or one week), according to the TSCV cycle. Figures 6 and 7 present heat map graphs for the DA forecast, revealing the importance of the input variables listed in Table 7. Figures 8 and 9 present heat map graphs for the WA forecast, revealing the importance of the input variables listed in Table 7. The analytical results obtained, as shown in the figures, can be summarized as follows. In the calendar data, we confirmed that the holiday was the most significant variable for the forecasting model, and the variables for the days of the week were highly important. The variables for the day did not significantly affect the model performance. We also confirmed that, overall, the temperature data were essential for the model performance, and the average temperature was a key input variable for the proposed model. In the historical load data, the electrical load from the previous day was the most important variable, and the electrical load one-week prior was also crucial for the DA forecasting model. In the WA forecasting models, the electrical load one-week prior was the most significant input variable in the historical load data. Here, we can see that the adjacent autocorrelation variables (e.g., historical load) are essential for the model performance. We also presume that the DA forecasting models performed better than the WA forecasting models because they could reflect the crucial historical load variables, both the day before and the week before.

### 3.5. Discussion

The experimental results showed that PLS exhibited poor prediction performance in TSCV. PLS is a popular method to deal with multicollinear relationships between output and input variables [48]. Even though we predicted the electrical load by setting the PLS hyperparameter automatically, it performed poorly. Although the historical load was highly correlated with the actual electrical load, PLS did not adequately reflect the historical load in TSCV, and hence we conclude that this caused the prediction accuracy to be low. XGB and CatBoost are state-of-the-art technologies. XGB performed satisfactorily, but CatBoost did not. Because most input variables are continuous, CatBoost could not use its advantages, such as ordered target statistics. Therefore, we concluded that XGB is better suited for DPLF and TDLF than CatBoost. The main advantage of the Cubist method is the addition of multiple training committees and “reinforcement” to balance the weights better. Therefore, we presume that Cubist can achieve satisfactory prediction performance because it predicts the next time point by adjusting the weights of the input variables better through TSCV.

The light GBM (LightGBM) and neural network methods also performed well in STLF but were not considered here. These methods require a sufficient dataset for model training [18, 60]; however, it takes a significant amount of time to collect enough data because only one dataset for daily peak load and total daily load is generated per day. Moreover, these methods are better configured for the Python environment [60]. In particular, neural network methods require high-performance computer specifications [9]. In this paper, we only considered several datasets collected over three years, and a little over 700 tuples were used for the first model training. Therefore, we did not expect these methods to formulate a robust forecasting model with such small datasets. In the future, we will apply these methods to perform interpretable hourly electrical load forecasting or VSTLF.

## 4. Conclusions

In this paper, we developed a novel forecasting model for interpretable short-term electrical load forecasting. To do this, we collected five different electrical load datasets with temperature and holiday information. We constructed different input variables by considering four forecasting types: day-ahead DPLF and TDLF and week-ahead DPLF and TDLF. We built the proposed model based on Cubist, a rule-based model, and applied TSCV to address the lack of data and reflect recent electrical load trends. The experimental results demonstrated that the proposed model showed excellent prediction performance. In addition, we conducted interpretable electrical load forecasting for each building or building cluster using the variable importance produced by the proposed model.

We found that applying the TSCV method can improve prediction performance, except for PLS, and that the Cubist method performed satisfactorily using a small dataset. It was challenging for CatBoost, a state-of-the-art technique, to produce excellent prediction performance because almost all input variables were configured as continuous. Overall, we confirmed that the higher the electrical load, the higher the prediction accuracy. TDLF and the day-ahead forecasting model had a better prediction performance than DPLF and the weak-ahead forecasting model. However, it was difficult to adequately train the forecasting models on sudden electrical load fluctuations because the amount of data was smaller than the amount of data for hourly load forecasting or VSTLF.

We plan to perform interpretable VSTLF, such as 10-minute or 15-minute-interval load forecasting, using neural network methods such as activation maps or an attention mechanism. In addition, we will make an effort to develop various methodologies for explainable forecasting in interpretable forecasting. We also plan to find variables that can reflect building characteristics and include them in the forecasting model.

## Figures and Tables

**Table 1:** Summary of recent STLF studies based on ML techniques.

Author (Year)	Dataset	Granularity	ML method	Rolling procedure	Model interpretability
Lee and Han [20] (2017)	South Korea provided by Korea Power Exchange (KPX)	Daily peak load	MLR	Yes	Yes
Fan et al. [21] (2019)	Australian Energy Market Operator (AEMO)	8 h	KNN	No	No
Dong et al. [22] (2021)	Qingdao City in China	1 h	Bagging	No	No
Sun et al. [23] (2021)	Tai'an City, Shandong Province in China	1 h	ANN	No	No
Truong et al. [24] (2021)	Residential building with a renewable energy system	1 h	AANN	No	No
Fan et al. [25] (2020)	New South Wales (NSW) in Australia	30 min	EMD SVR PSO AR-GARCH	No	Yes
Zhang et al. [26] (2020)	Queensland (QLD) in Australia	30 min	VMD SR SVR CBCS	Yes	No
Bouktif et al. [27] (2018)	Metropolitan France	30 min	GA LSTM-RNN	Yes	Yes
Wang et al. [28] (2018)	University campus in Florida	1 h	RF	No	Yes
Ruiz-Abellón et al. [29] (2018)	University campus in Spain	1 h	Bagging RF CRF XGB	No	Yes
Abbasi et al. [30] (2019)	AEMO	30 min	XGB	No	Yes
Zhang et al. [31] (2020)	More than 1,400 enterprises in Yangzhong High-Tech Zone, China	Daily	K-means clustering CatBoost	No	Yes

**Table 2:** Building information.

Dataset #	Number of buildings	Building type (description)	Location	Dataset period	Public access
Building 1	1	Commercial (office)	Richland, Washington	Jan. 2, 2009–Dec. 31, 2011	Yes
Building 2	1	Commercial (office)	Richland, Washington	Jan. 2, 2009–Dec. 31, 2011	Yes
Cluster 1	16	Educational (dormitory)	Seoul, South Korea	Jan. 1, 2016–Dec. 31, 2018	No
Cluster 2	32	Educational (humanities bldg.)	Seoul, South Korea	Jan. 1, 2016–Dec. 31, 2018	No
Cluster 3	5	Educational (engineering bldg.)	Seoul, South Korea	Jan. 1, 2016–Dec. 31, 2018	No

**Table 3:** Statistics on daily peak electrical load data (unit: kW).

Statistics	Building 1	Building 2	Cluster 1	Cluster 2	Cluster 3
Number of valid cases	1094	1094	1096	1096	1096
Mean	49.16	54.46	1575.94	4132.02	2606.01
Standard deviation	21.69	21.15	308.91	1327.07	451.57
Trimmed mean	50.40	56.23	1552.32	4325.76	2623.04
Median	48.59	54.52	1561.97	4176.24	2670.00
Median absolute deviation	19.87	18.52	321.31	1537.16	437.66
Minimum	8.86	10.97	878.40	1426.56	1579.20
Maximum	141.11	135.00	2623.68	6900.48	3549.60
Range	132.25	124.03	1745.28	5473.92	1970.40
Skew	0.34	0.05	0.42	–0.25	–0.31
Kurtosis	0.43	0.09	–0.15	–1.03	–0.71
Standard error	0.66	0.64	9.33	40.09	13.66

**Table 4:** Statistics on total daily electrical load data (unit: kW).

Statistics	Building 1	Building 2	Cluster 1	Cluster 2	Cluster 3
Number of valid cases	1094	1094	1096	1096	1096
Mean	719.31	852.63	29802.41	62563.10	49440.52
Standard deviation	250.53	290.95	5350.39	17167.95	6409.23
Trimmed mean	723.27	850.15	29390.76	62514.48	49506.54
Median	714.88	844.97	29569.92	62872.23	49771.80
Median absolute deviation	229.40	244.27	5407.28	20269.87	6802.47
Minimum	198.22	242.09	19013.04	27961.44	32546.40
Maximum	1527.30	2130.50	49235.76	98475.84	64403.70
Range	1329.08	1888.41	30222.72	70514.40	31857.30
Skew	0.16	0.44	0.45	–0.13	–0.11
Kurtosis	–0.32	0.98	–0.03	–1.06	–0.64
Standard error	7.57	8.80	161.61	518.58	193.86

**Table 5:** Residual standard error and R-squared statistics for daily peak electrical load data.

Statistics	Building 1		Building 2		Cluster 1		Cluster 2		Cluster 3	
	1D	2D	1D	2D	1D	2D	1D	2D	1D	2D
Residual standard error (unit: kW)	17.7	17.1	17.4	16.5	285.8	271.4	1128	1121	365.9	351.9
Multiple R-squared (unit: %)	33.9	38.6	32.6	39.7	14.6	23.2	27.9	29.1	36.4	41.3
Adjusted R-squared (unit: %)	33.7	38.2	32.4	39.4	14.4	22.8	27.7	28.7	36.2	41.0

**Table 6:** Residual standard error and R-squared statistics for total daily electrical load data.

Statistics	Building 1		Building 2		Cluster 1		Cluster 2		Cluster 3	
	1D	2D	1D	2D	1D	2D	1D	2D	1D	2D
Residual standard error (unit: kW)	211	203	256	228	4958	4698	14680	14670	5699	5417
Multiple R-squared (unit: %)	29.3	34.9	22.9	39.0	14.4	23.3	27.1	27.4	27.4	34.6
Adjusted R-squared (unit: %)	29.1	34.6	22.7	38.7	14.1	22.9	26.9	27.0	27.2	34.2

**Table 7:** Input variables for day-ahead and week-ahead forecasts.

IV #	Input variables for day-ahead forecasting	Input variables for week-ahead forecasting	Variable type
01	Month_x_	Month_x_	Continuous [–1, 1]
02	Month_y_	Month_y_	Continuous [–1, 1]
03	Day_x_	Day_x_	Continuous [–1, 1]
04	Day_y_	Day_y_	Continuous [–1, 1]
05	Day of the week_x_	Day of the week_x_	Continuous [–1, 1]
06	Day of the week_y_	Day of the week_y_	Continuous [–1, 1]
07	Holiday	Holiday	Binary
08	Minimum temperature	Minimum temperature	Continuous
09	Average temperature	Average temperature	Continuous
10	Maximum temperature	Maximum temperature	Continuous
11	Holiday (the day before seven days)	Holiday (the day before four weeks)	Binary
12	Electrical load (the day before seven days)	Electrical load (the day before four weeks)	Continuous
13	Holiday (the day before six days days)	Holiday (the day before six three weeks)	Binary
14	Electrical load (the day before six days)	Electrical load (the day before three weeks)	Continuous
15	Holiday (the day before five days)	Holiday (the day before five two weeks)	Binary
16	Electrical load (the day before five days)	Electrical load (the day before two weeks)	Continuous
17	Holiday (the day before four days)	Holiday (the day before one week)	Binary
18	Electrical load (the day before four days)	Electrical load (the day before one week)	Continuous
19	Holiday (the day before three days)	—	Binary
20	Electrical load (the day before three days)	—	Continuous
21	Holiday (the day before two days)	—	Binary
21	Electrical load (the day before two days)	—	Continuous
23	Holiday (the day before one day)	—	Binary
24	Electrical load (the day before one day)	—	Continuous

**Table 8:** List of the hyperparameters used to build optimal forecasting models.

Methods	Package	Hyperparameters and their range
MLR [53]	lm	None (automatic identification)
PLS [53]	pls, caret	ncomp (vector of positive integers): 1 : 1 less than the number of input variables
MARS [49]	earth, caret	degree (maximum degree of interactions): 1 : 3
		nprune (number of terms retained in the final model): 2, 13, 24, 35, 46, 56, 67, 78, 89, 100
KNN [21]	caret	k (number of neighbors): 2
SVR [54]	kernlab, caret	sigma (sigma): 0.35, 0.4, 0.1
		C (cost): 1, 3, 5, 8, 10, 12
DT [53]	rpart	maxdepth (maximum depth of any node of the final tree): automatic identification
Bagging [53]	Ipred	None (automatic identification)
RF [40, 48]	randomForest	mtry (number of variables randomly chosen at each split): number of input variables divided by 3
		ntree (number of trees to grow): 128
GBM [55]	gbm	n.trees (number of trees to grow): 3000, 4000, 5000, 6000, 7000, 8000, 9000, 10000
		interaction.depth (maximum depth of each tree): 5
		shrinkage (shrinkage or learning rate parameter): 0.001
		bag.fraction (subsampling rate): 0.5
XGB [56]	xgboost, caret	nrounds (number of trees to grow): 50, 100, 250, 500
		eta (shrinkage or learning rate parameter): 0.01, 0.1, 1
		lambda (L2 regularization term on weights): 0.1, 0.5, 1
		alpha (L1 regularization term on weights): 0.1, 0.5, 1
CatBoost [57]	catboost, caret	learning rate (shrinkage or learning rate parameter): 0.03, 0.1
		depth (maximum depth of each tree): 4, 6, 10
		l2_leaf_reg (coefficient at the L2 regularization term of the cost function): 1, 3, 5, 7, 9
Cubist [50]	Cubist	committees (sequence generation of rule-based models (similar to boosting)): 1, 10, 50, 100
		neighbors (single integer value to adjust the rule-based predictions from the training set): 0, 1, 5, 9

**Table 9:** MAPE comparison of DA-DPLF (%).

Methods	Building 1		Building 2		Cluster 1		Cluster 2		Cluster 3	
	Holdout	TSCV	Holdout	TSCV	Holdout	TSCV	Holdout	TSCV	Holdout	TSCV
MLR	21.15	20.88	18.27	17.95	5.55	5.55	8.91	8.95	3.68	3.81
PLS	21.10	33.30	18.45	28.21	5.51	10.77	8.95	22.90	3.70	9.15
MARS	20.32	18.83	18.08	16.28	4.96	4.70	5.90	5.95	2.83	2.87
KNN	22.35	22.15	19.97	19.49	6.65	6.02	9.49	8.80	3.98	3.87
SVR	22.17	20.82	17.37	15.95	5.94	**4.66**	7.42	6.56	2.81	**2.29**
DT	22.11	23.20	26.04	23.10	8.71	8.05	12.07	11.56	5.22	5.05
Bagging	23.08	22.11	22.13	20.56	7.30	6.58	10.91	10.39	4.67	4.43
RF	19.72	17.78	18.42	15.42	5.90	5.12	6.45	5.99	2.98	2.63
GBM	18.17	16.99	17.90	15.38	5.42	4.87	6.21	5.82	2.99	2.71
XGB	19.93	17.17	18.50	15.08	6.23	5.32	6.25	5.70	2.94	2.68
CatBoost	22.61	20.31	19.31	16.88	6.49	5.49	7.48	6.65	3.58	3.05
Cubist	18.60	**16.98**	14.97	**13.51**	4.90	4.68	5.09	**5.03**	3.15	2.78

Values in bold indicate the lowest values for the respective datasets.

**Table 10:** CVRMSE comparison of DA-DPLF (%).

Methods	Building 1		Building 2		Cluster 1		Cluster 2		Cluster 3	
	Holdout	TSCV	Holdout	TSCV	Holdout	TSCV	Holdout	TSCV	Holdout	TSCV
MLR	24.03	23.85	19.35	19.18	7.36	7.35	10.03	10.00	5.50	5.85
PLS	24.07	34.33	19.47	27.73	7.31	12.83	10.04	22.38	5.50	11.38
MARS	28.66	23.11	18.47	17.88	6.94	6.45	6.83	6.62	3.41	3.71
KNN	29.75	27.70	22.28	22.15	9.71	8.70	12.05	11.06	5.42	5.30
SVR	30.28	26.61	17.65	17.44	11.06	7.66	8.78	7.66	4.53	3.99
DT	26.34	26.86	24.73	22.67	12.28	10.66	12.61	12.49	5.81	5.62
Bagging	26.90	25.18	20.54	19.59	10.81	9.01	11.04	10.71	5.27	5.00
RF	27.93	23.33	18.26	17.03	8.81	7.19	7.09	6.55	3.57	**3.24**
GBM	26.68	**22.87**	18.08	16.92	8.07	6.75	6.91	6.44	3.53	3.28
XGB	29.37	23.13	18.43	17.60	9.32	7.27	7.28	6.65	3.56	3.32
CatBoost	31.20	26.08	19.48	18.07	9.45	7.56	8.09	7.31	4.35	3.90
Cubist	26.90	23.22	17.34	**16.16**	6.72	**6.29**	**5.63**	5.67	4.45	3.62

Values in bold indicate the lowest values for the respective datasets.

**Table 11:** MAPE comparison of DA-TDLF (%).

Methods	Building 1		Building 2		Cluster 1		Cluster 2		Cluster 3	
	Holdout	TSCV	Holdout	TSCV	Holdout	TSCV	Holdout	TSCV	Holdout	TSCV
MLR	11.18	11.12	10.98	10.66	3.61	3.63	7.06	7.11	2.55	2.73
PLS	11.18	26.27	11.10	27.34	3.60	8.62	7.03	18.13	2.57	7.49
MARS	9.39	**8.64**	11.59	9.97	3.28	3.36	4.30	4.26	2.08	2.09
KNN	13.03	12.05	18.82	17.67	6.00	5.05	7.42	6.89	4.79	4.63
SVR	9.84	8.74	17.14	10.74	3.97	**2.61**	5.19	4.46	2.89	2.15
DT	17.40	17.29	23.68	19.71	6.96	6.16	9.12	8.86	4.72	4.22
Bagging	15.89	15.24	18.90	16.11	5.80	5.01	8.33	8.00	4.18	3.48
RF	10.92	9.95	15.01	9.58	4.44	3.97	4.51	4.29	2.46	2.05
GBM	10.74	9.77	14.44	10.04	4.08	3.59	4.61	4.42	2.50	2.08
XGB	10.98	9.86	13.66	9.47	4.54	3.84	4.54	4.38	2.63	2.18
CatBoost	11.49	10.20	14.94	10.07	4.92	3.81	5.27	4.70	2.95	2.23
Cubist	8.80	8.89	11.20	**8.39**	3.26	3.24	**3.55**	3.60	2.36	**1.98**

Values in bold indicate the lowest values for the respective datasets.

**Table 12:** CVRMSE comparison of DA-TDLF (%).

Methods	Building 1		Building 2		Cluster 1		Cluster 2		Cluster 3	
	Holdout	TSCV	Holdout	TSCV	Holdout	TSCV	Holdout	TSCV	Holdout	TSCV
MLR	14.23	14.17	12.98	12.79	4.84	4.83	8.31	8.31	4.43	4.45
PLS	14.13	26.59	12.95	25.95	4.82	10.19	8.31	19.07	4.45	9.14
MARS	12.59	**11.02**	12.85	11.60	4.44	4.39	4.99	5.00	3.44	3.82
KNN	17.23	16.73	19.36	18.61	8.41	6.97	10.37	9.65	6.57	6.45
SVR	12.45	11.80	16.67	12.56	8.67	4.84	7.12	6.05	5.54	5.04
DT	22.07	22.38	23.08	21.57	9.61	8.38	10.41	10.46	5.41	4.95
Bagging	19.91	19.96	18.19	17.53	8.37	7.05	9.57	9.19	4.73	4.04
RF	14.48	13.59	14.89	11.68	6.86	5.65	5.43	5.16	2.95	**2.66**
GBM	13.47	12.86	14.54	12.17	6.25	5.10	5.56	5.31	2.94	2.79
XGB	14.86	13.77	14.71	12.45	7.00	5.45	5.65	5.48	3.12	3.66
CatBoost	14.34	13.52	15.07	12.34	7.34	5.36	6.74	5.86	3.57	2.85
Cubist	12.09	11.72	13.16	**10.55**	4.46	**4.36**	**4.15**	4.24	3.27	3.34

Values in bold indicate the lowest values for the respective datasets.

**Table 13:** MAPE comparison of WA-DPLF (%).

Datasets	Evaluation	Forecasting methods											
		MLR	PLS	MARS	KNN	SVR	DT	Bagging	RF	GBM	XGB	CatBoost	Cubist
Building 1	Holdout	25.89	26.25	22.15	24.11	20.03	26.97	24.64	19.92	19.44	19.45	21.15	20.68
	TSCV (avg.)	26.08	28.62	21.06	23.54	19.36	25.58	23.59	19.48	19.16	19.00	20.15	19.13

Building 2	Holdout	21.52	21.60	19.28	21.97	18.92	24.82	22.04	18.60	18.31	17.44	18.76	16.92
	TSCV (avg.)	21.26	23.89	17.10	21.17	16.99	23.58	20.97	15.87	15.56	15.65	16.87	14.55

Cluster 1	Holdout	8.00	8.01	6.82	8.53	7.91	9.60	8.89	7.89	7.55	8.25	7.89	6.77
	TSCV (avg.)	8.16	10.47	7.01	8.07	6.80	9.47	8.71	7.26	6.99	7.77	7.26	6.53

Cluster 2	Holdout	11.27	11.38	8.75	9.79	8.11	12.48	11.49	7.75	7.60	7.87	8.48	7.04
	TSCV (avg.)	11.18	18.27	7.32	9.88	7.56	11.92	11.07	7.46	7.18	7.41	8.24	6.87

Cluster 3	Holdout	4.77	4.85	3.82	4.86	3.14	5.76	5.24	3.50	3.53	3.57	3.93	3.40
	TSCV (avg.)	4.93	7.38	3.57	4.63	2.83	5.56	4.94	3.19	3.22	3.43	3.57	3.25

**Table 14:** CVRMSE comparison of WA-DPLF (%).

Datasets	Evaluation	Forecasting methods											
		MLR	PLS	MARS	KNN	SVR	DT	Bagging	RF	GBM	XGB	CatBoost	Cubist
Building 1	Holdout	31.49	31.55	48.77	33.57	32.38	34.54	32.32	31.44	29.74	32.20	32.90	30.09
	TSCV (avg.)	31.40	35.83	39.15	32.88	29.03	32.16	29.13	27.82	27.40	26.73	29.61	27.44

Building 2	Holdout	23.31	23.36	19.83	23.62	19.77	23.07	20.54	19.09	18.93	19.51	20.17	19.02
	TSCV (avg.)	23.04	27.03	18.99	22.79	19.43	23.84	19.98	18.23	17.50	18.42	19.31	18.07

Cluster 1	Holdout	10.89	10.89	9.21	11.97	11.94	13.40	12.45	11.05	10.57	11.69	10.98	9.17
	TSCV (avg.)	10.82	13.66	9.28	11.39	9.81	12.76	11.77	10.02	9.57	10.59	9.87	8.92

Cluster 2	Holdout	12.13	12.19	9.60	11.94	9.11	13.29	11.95	8.47	8.57	9.36	9.41	8.08
	TSCV (avg.)	12.18	20.30	8.35	12.10	8.57	12.90	11.60	8.29	8.24	8.70	9.00	8.00

Cluster 3	Holdout	6.61	6.47	4.58	5.93	4.21	6.33	5.80	4.12	4.12	4.25	4.92	4.14
	TSCV (avg.)	7.15	10.07	4.44	5.69	3.89	6.13	5.50	3.94	3.87	4.21	4.43	4.01

**Table 15:** MAPE comparison of WA-TDLF (%).

Datasets	Evaluation	Forecasting methods											
		MLR	PLS	MARS	KNN	SVR	DT	Bagging	RF	GBM	XGB	CatBoost	Cubist
Building 1	Holdout	17.18	17.61	12.68	16.02	12.51	20.41	16.69	12.56	12.45	13.37	13.31	11.62
	TSCV (avg.)	17.23	18.35	12.05	15.11	11.96	18.40	15.95	11.80	11.87	12.05	12.54	11.46

Building 2	Holdout	17.47	17.86	15.77	21.78	17.01	24.42	19.64	16.28	16.45	15.13	16.98	15.24
	TSCV (avg.)	16.19	16.80	12.95	20.37	11.28	17.82	15.59	10.82	11.88	10.85	12.57	10.94

Cluster 1	Holdout	6.76	6.87	6.11	8.58	6.70	9.03	7.73	7.04	6.61	7.08	7.14	5.90
	TSCV (avg.)	6.90	9.05	5.71	8.01	5.49	8.76	7.82	6.46	6.05	6.54	6.25	5.51

Cluster 2	Holdout	9.73	9.92	5.98	7.90	6.18	9.79	9.13	5.89	5.57	5.85	6.47	4.91
	TSCV (avg.)	9.84	15.07	5.63	7.76	5.63	9.81	9.18	5.86	5.53	5.67	6.15	5.11

Cluster 3	Holdout	4.51	4.54	3.45	5.97	3.88	5.40	4.79	3.20	3.28	3.29	3.63	2.96
	TSCV (avg.)	4.76	5.97	3.05	5.56	3.62	4.87	4.24	2.75	2.86	2.91	3.06	2.88

**Table 16:** CVRMSE comparison of WA-TDLF (%).

Datasets	Evaluation	Forecasting methods											
		MLR	PLS	MARS	KNN	SVR	DT	Bagging	RF	GBM	XGB	CatBoost	Cubist
Building 1	Holdout	21.71	22.25	15.16	19.58	15.71	24.91	20.65	16.35	15.10	16.57	17.49	15.04
	TSCV (avg.)	21.59	25.73	14.18	18.26	15.33	22.97	20.26	15.43	14.82	15.73	16.33	14.82

Building 2	Holdout	19.73	20.43	16.75	22.77	17.00	23.67	19.69	16.15	16.77	15.88	17.20	16.33
	TSCV (avg.)	19.27	22.77	14.39	21.44	13.95	20.83	17.68	13.73	13.98	14.21	15.42	13.41

Cluster 1	Holdout	9.21	9.27	7.82	11.24	10.24	12.06	10.60	9.57	8.93	9.84	9.67	7.78
	TSCV (avg.)	9.17	11.85	7.39	10.60	7.94	11.47	10.52	8.62	8.08	8.73	8.30	7.39

Cluster 2	Holdout	11.35	11.43	7.47	10.80	8.04	11.46	10.16	6.93	6.72	7.37	7.82	6.08
	TSCV (avg.)	11.55	17.95	6.63	10.62	7.18	11.48	10.45	6.94	6.74	7.23	7.47	6.27

Cluster 3	Holdout	6.48	6.24	4.19	7.78	6.55	5.96	5.32	3.82	3.82	3.94	4.35	3.68
	TSCV (avg.)	7.43	7.83	3.95	7.49	6.40	5.69	4.89	3.41	3.44	3.72	3.82	3.65

**Table 17:** Ranks of each model based on the performance metrics and average rank.

Methods	MAPE				CVRMSE				Average rank
	DA-DPLF	DA-TDLF	WA-DPLF	WA-TDLF	DA-DPLF	DA-TDLF	WA-DPLF	WA-TDLF	
MLR	8.2	7.4	9.8	9.4	7.8	7	9.4	9.6	8.6
PLS	12	12	12	11.4	12	12	11.8	12	11.9
MARS	4.8	2.8	5.4	4.6	3.8	2.6	6	3.2	4.2
KNN	9	9.6	8.2	9.8	9.8	9.8	9.2	9.8	9.4
SVR	4	4.4	3.8	3.8	7	6.2	4.6	4.8	4.8
DT	11	10.8	10.8	10.8	10.6	10.6	10.4	10	10.6
Bagging	9.8	9.4	9.2	8.6	8.6	9	8	8.2	8.9
RF	4	4.4	4.2	3.2	3.2	4.2	3.8	3.6	3.8
GBM	3.2	4.2	2.6	3.2	**2**	3.8	**1.8**	3	3.0
XGB	3.2	4.8	4	4.6	4.2	6	4.6	5.6	4.6
CatBoost	6.8	6.6	5.8	6.2	6.8	5	6.2	6.2	6.2
Cubist	**2**	**1.6**	**1.8**	**2**	2.2	**1.8**	2.2	**1.6**	**1.9**

Values in bold indicate the lowest values for the respective electrical load forecasting types (DA: day-ahead; WA: week-ahead).

**Table 18:** Prediction performance of Lee and Han's MLR and Cubist.

Datasets	Metrics	DPLF		TDLF	
		MLR [20]	Cubist	MLR [20]	Cubist
Building 1	MAPE	28.98	16.98	17.19	8.89
	CVRMSE	29.58	23.22	19.57	11.72

Building 2	MAPE	24.44	13.51	16.05	8.39
	CVRMSE	24.57	16.16	17.82	10.55

Cluster 1	MAPE	7.22	4.68	5.00	3.24
	CVRMSE	9.17	6.29	6.49	4.36

Cluster 2	MAPE	13.62	5.03	16.59	3.60
	CVRMSE	10.22	5.67	13.66	4.24

Cluster 3	MAPE	6.20	2.78	5.12	1.98
	CVRMSE	8.83	3.62	7.71	3.34

**Table 19:** Paired sample *t*-test of holdout and TSCV.

Statistics	MAPE	CVRMSE
*T*-test statistic value (t)	11.136	11.167
Degrees of freedom (df)	219	219
Significance level of the *t*-test (*p* value)	2.2 × 10^−16^	2.2 × 10^−16^
Confidence interval (conf.int) of the mean differences at 95%	[0.787, 1.125]	[0.821, 1.173]
Mean differences between pairs (sample estimates)	0.956	0.997

**Table 20:** Results of Wilcoxon signed-rank and Friedman tests.

Methods	Wilcoxon signed-rank test		Friedman test	
	MAPE	CVRMSE	MAPE	CVRMSE
MLR	1.907 × 10^−6^	1.907 × 10^−6^	2.2 × 10^−16^	2.2 × 10^−16^
PLS	1.907 × 10^−6^	1.907 × 10^−6^		
MARS	3.624 × 10^−5^	0.005841		
KNN	1.907 × 10^−6^	1.907 × 10^−6^		
SVR	0.009463	5.722 × 10^−6^		
DT	1.907 × 10^−6^	1.907 × 10^−6^		
Bagging	1.907 × 10^−6^	1.907 × 10^−6^		
RF	0.000168	0.001432		
GBM	8.202 × 10^−5^	0.019580		
XGB	6.294 × 10^−5^	0.000210		
CatBoost	1.907 × 10^−6^	0.000175		

## Data Availability

The data that support the findings of this study are available from https://www.dropbox.com/s/y0bcrulfqywra1x/Datasets.zip?dl = 0, but restrictions apply to the availability of these data.

## Abbreviations

1D: One-dimensional
2D: Two-dimensional
AANN: Additive artificial neural network
ARIMA: Autoregressive integrated moving average
ARMA: Autoregressive moving average
ANN: Artificial neural network
Bagging: Bootstrap aggregating
BEMS: Building energy management system
CatBoost: Categorical boosting
CBCS: Cuckoo bird search process of the cuckoo search algorithm
CRF: Conditional random forest
CVRMSE: Coefficient of variation of the root mean square error
DA: Day-ahead
DPLF: Daily peak load forecasting
DT: Decision tree
EMD: Empirical mode decomposition
ESS: Energy storage system
ET: Extra tree
GBM: Gradient boosting machine
GHG: Greenhouse gas
IMF: Intrinsic mode function
KNN: K-nearest neighbor
KPX: Korea Power Exchange
LightGBM: Light gradient boosting machine
LSTM: Long short-term memory
MAPE: Mean absolute percentage error
MARS: Multivariate adaptive regression splines
MLR: Multiple linear regression
PLS: Partial least squares
PSO: Particle swarm optimization
RE: Renewable energy
RF: Random forest
SR: Self-recurrent
STLF: Short-term load forecasting
SVR: Support vector regression
TDLF: Total daily load forecasting
TSCV: Time-series cross-validation
VSTLF: Very short-term load forecasting
WA: Week-ahead
XGB: Extreme gradient boosting.
